# Modeling of and Experimenting with Concentric Tube Robots: Considering Clearance, Friction and Torsion

**DOI:** 10.3390/s23073709

**Published:** 2023-04-03

**Authors:** Tianxiang Liu, Gang Zhang, Peng Zhang, Tianyu Cheng, Zijie Luo, Shengsong Wang, Fuxin Du

**Affiliations:** 1 School of Mechanical Engineering, Shandong University, Jinan 250061, China; 2 Key Laboratory of High-Efficiency and Clean Mechanical Manufacture of MOE, Shandong University, Jinan 250061, China; 3 Engineering Research Center of Intelligent Unmanned System, Ministry of Education, Jinan 250061, China; 4 Shandong Center for Food and Drug Evaluation & Inspection, Jinan 250014, China

**Keywords:** concentric tube robots, database, frictional analysis, compensation, modeling

## Abstract

Concentric tube robots (CTRs) are a promising prospect for minimally invasive surgery due to their inherent compliance and ability to navigate in constrained environments. Existing mechanics-based kinematic models typically neglect friction, clearance, and torsion between each pair of contacting tubes, leading to large positioning errors in medical applications. In this paper, an improved kinematic modeling method is developed. The effect of clearance on tip position during concentric tube assembly is compensated by the database method. The new kinematic model is mechanic-based, and the impact of friction moment and torsion on tubes is considered. Integrating the infinitesimal torsion of the concentric tube robots eliminates the errors caused by the interaction force between the tubes. A prototype is built, and several experiments with kinematic models are designed. The results indicate that the error of tube rotations is less than 2 mm. The maximum error of the feeding experiment does not exceed 0.4 mm. The error of the new modeling method is lower than that of the previous kinematic model. This paper has substantial implications for the high-precision and real-time control of concentric tube robots.

## 1. Introduction

In the last decade, robot-assisted minimally invasive surgery (RAMIS) has evolved rapidly, with more than 60 emerging RAMIS robot types being developed. The most widespread surgical robot system has been the da Vinci robot system, which has been used in thoracic surgery, gastrointestinal tract, and laparoscopy [1,2,3,4]. Now, RAMIS is developing in the direction of miniaturization and intelligence, etc. [5].

To provide the best treatment, especially for deep pathologies, minimally invasive surgery requires instrumentation with increased dexterity and flexibility to overcome the challenges of a confined surgical workspace and lack of articulation. Conventional robots have mechanical rigidity and difficulty controlling their interaction with organs. Continuum robots with greater flexibility have received attention in the field of minimally invasive surgery [6].

Continuum robot designs can be classified predominantly by the method of shape control [7]: multi-backbone designs [8], tendon-actuated designs [9,10,11], shape memory designs [12], and soft robot designs [13,14,15]. Several hybrid driving schemes have also been applied, including pneumatic muscles with embedded elastic rods [16], and the system combining the concentric tube robot and the notched continuum robot [17].

Concentric tube robots are a typical class of continuous robots [18]. They are semi-rigid, and have a small size and flexible bending, allowing them to perform complex operations in the natural cavity and resist interference [19,20,21,22]. Currently, concentric tube robots are used in cardiac interventions [23], skull base tumor resection [24], and lung puncture [25].

The classical concentric tube model establishes the relationship between the curvature and bending moment, and uses the moment equilibrium equation to derive the curvature after nesting. The classical kinematic models neglect the influence of friction, torsion, and clearance on the end position of concentric tube robots. After the concentric tube robot is assembled, twisting exists between each pair of contact tubes. Neglecting the relative twist causes a tip position error of about 10% [26,27]. In addition, there are clearances between the tubes, which will cause a positioning error at the end of the concentric tube robots.

Dupont et al. have developed a kinematic model for concentric tube robots, considering torsion and friction. Although this modeling method effectively reduces the prediction error of the kinematic model for the tip position, it does not consider the effect caused by clearance on the tip position of concentric tube robots. J. Ha’s team incorporated clearance and friction into a kinematic model, and developed a friction model for concentric tube robots based on the small clearance assumption [28]. Compared with the classical kinematic model of the concentric tube robot, the maximum error of the concentric tube robot’s tip position is reduced by half. Nonetheless, it has a complex modeling process, and the calculation time is prolonged. This model is used for path planning, and not real-time control.

In this paper, we have added friction, torsion, and clearance to the kinematic model. The tube’s curvature is affected by torsion and friction, so the constant curvature assumption no longer applies. In the new method, the concentric tube robot is segmented into infinitesimal segments, and the twist is integrated in each segment. When the tubes are assembled, a gap is produced, causing additional deflection of the tubes. The deflection angle can be derived from experimental data.

The contributions of this paper are as follows:

1. A new kinematic model of concentric tube robots based on the variable curvature assumption and the infinitesimal method is developed. The new model considers three effects on the tip position of CTRs: friction, torsion, and clearance. The model can predict the tip position accurately;
2. The kinematic modeling process is simplified. Compared with the existing modeling methods that consider clearance, friction, and torsion, the new approach changes the order in which the three effects are modeled. The improved modeling is concise and allows for faster modeling;
3. Forward kinematic experiments are designed to verify the accuracy of the improved model. These prove that the accuracy of the improved model is much higher than that of the previous one, which neglects clearance, torsion, and friction.

The rest of the paper is as follows: Section 2 presents the effects of clearance and torsion on concentric tube robots. The improved kinematic model is derived in Section 3. Section 4 conducts kinematic experiments of the concentric tube robots to validate the new kinematic model, and conclusions are presented in Section 5.

## 2. Problem Statement

In the past few years, some progress has been made in the kinematics modeling of the concentric tube robot, but some aspects still need to be improved. There are contact forces between the tubes, and the concentric tube robot is not a rigid body. When there is a relative translation and rotation between the tubes, friction forces and torques are generated, affecting the torsion angle between the tubes and leading to a more complex path of the tube tips.

**Figure 1:**
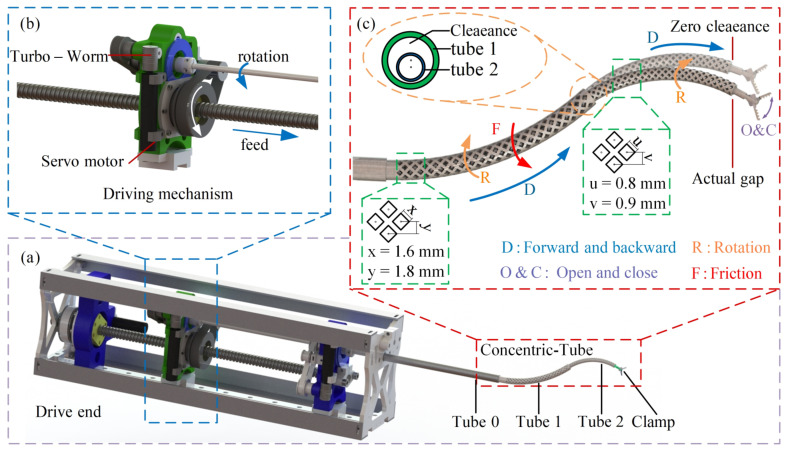
Concentric tube robots: (**a**) overall schematic diagram of concentric tube robots, (**b**) drive module, (**c**) degrees of freedom and friction.

To carry out further research, a prototype was designed, as shown in Figure 1. The two concentric tubes have been pre-bent. A motor powers the rotation and feeding of the tube, and the rotation is belt driven; the feeding is achieved by a worm gear mechanism, as shown in Figure 1b. With future commercialization in mind, the structure of most parts of the prototype was optimized to reduce costs during mass production [29].

Due to the clearance between the inner and outer tubes, when the two tubes of the concentric tube robot are assembled, if the curvature of the two tubes is not equal, the centerline of the inner tube will deviate from that of the outer one. As shown in Figure 1, this phenomenon can be considered the additional deflection of the inner tube, leading to a non-negligible tip positioning error.

The constitutive and static equilibrium differential equations are traditional methods to establish the forward kinematics model of concentric tube robots. These methods are based on the assumption of constant curvature and torsional rigidity. These models cannot simulate the existence of torsion and clearance in concentric tube robots, and the prediction ability for the actual tip position of concentric tube robots is poor. Therefore, it cannot meet the requirements for medical applications. In existing studies, models considering these three factors can only be used for path planning due to their complexity and slow computational speed.

Above all, a modified kinematic model of concentric tubes has been devised. In the new modeling method, torsion and friction are incorporated into the model first. Furthermore, the clearance data are added to the model. It also has a simpler kinematic modeling process and less computation time.

## 3. Kinematic Model Considering the Frictional Clearance

### 3.1. Curvature Solution Considering Concentrated Frictional Moment

**Figure 2:**
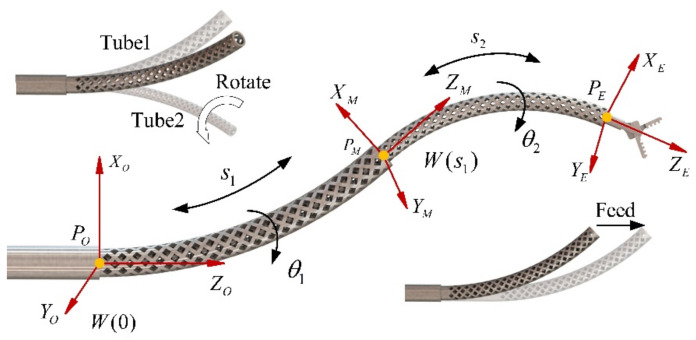
Coordinate system establishment.

The concentric tube robotic system has four DOFs: *s*_1_ refers to the feed of the outer tube, *θ*_1_ is the rotation angle around the *z*-axis, and *s*_2_ and *θ*_2_ are the feed and rotation angles of the inner tube, respectively. Establishing the coordinate system, as shown in Figure 2, *W*(0) is the world coordinate system of the initial point. The coordinate system *W*(*s*) can be obtained by sliding this coordinate system along the center line of the tube without rotation to the arc length *s*. When rotating *W*(*s*) by an angle *θ*_1_, the tube’s outer coordinate system *B*_1_(s) can be obtained. Similarly, *B*_2_(*s*) of the inner tube can be obtained. *P_M_* and *P_E_* are the tip positions of the outer tube and inner tube of the concentric tube robots. The rigid straight tube is not affected by the relative rotation between the two tubes. However, the relative rotation leads to the inner and outer tubes having the same curvature during assembling.

Suppose the curvature *u_i_*(*s*) of each section of the concentric tube robot at arc length *s* is expressed as

(1) $$u_{i}\left(s\right)={\left[u_{ix}\left(s\right),u_{iy}\left(s\right),u_{iz}\left(s\right)\right]}^{Τ}$$

where *u_ix_*(*s*), *u_iy_*(*s*), and *u_iz_*(*s*) are the components of the curvature along the *x*, *y*, and *z* directions, respectively.

The bending moment $M_{i}^{B_{i}\left(s\right)}\left(s\right)$ of tube *i* due to the relative rotation of any section of the concentric tube can be expressed as

(2) $$M_{i}^{B_{i}\left(s\right)}\left(s\right)=K_{i}\left(u_{i}^{B_{i}\left(s\right)}\left(s\right)-{\bar{u}}_{i}^{B_{i}\left(s\right)}\left(s\right)\right)$$

where $u_{i}^{B_{i}\left(s\right)}(s)$ and ${\bar{u}}_{i}^{B_{i}\left(s\right)}(s)$ are denoted as the curvature in the coordinate system *B_i_*(*s*) and the initial curvature, respectively; *K_i_* is the stiffness tensor independent of the coordinate system; *k_ix_* and *k_iy_* are the magnitudes of stiffness in the *x* and *y* directions, respectively; and *k_iz_* is the torsional stiffness in the *z* direction. *K_i_* can be expressed as

(3) $$K_{i}=\left[\begin{array}{ccc} k_{ix} & 0 & 0\\ 0 & k_{iy} & 0\\ 0 & 0 & k_{iz} \end{array}\right]$$

During the inner and outer tube rotation, the concentrated frictional moment generated by the concentrated bending moment causes the adjacent tubes to twist, resulting in an inevitable loss when the rotation angle *θ_i_* is transmitted along the tube length direction. In this paper, considering the slight torsional stiffness of the concentric tube robots, the transmission loss is not negligible. Since torsion causes additional bending of the concentric tube, the curvature of the mating segment is a function of the arc length s [30].

Considering only the case of two tubes, the actual relative angle is *β*(*s*), which can be expressed as

(4) $$\beta \left(s\right)=\theta_{2}\left(s\right)-\theta_{1}\left(s\right)$$

The moment balance equation in the fit section and the compatibility equation for concentric tube robots under torsion can be rewritten as

(5) $$M_{1}^{B_{1}\left(s\right)}-R_{z}\left(\beta \left(s\right)\right)M_{2}^{B_{2}\left(s\right)}=0$$

(6) $$u_{1}^{B_{1}\left(s\right)}\left(s\right)=R_{z}\left(\beta \left(s\right)\right)u_{2}^{B_{2}\left(s\right)}\left(s\right)-\dot{\beta }\left(s\right)e_{z}$$

where *e_z_* = [0, 0, 1] ^T^ and $\dot{\beta }=d\beta /ds$. This equation ensures that the tube has the same curvature in the cross-sectional plane but allows for different torsional rates. By combining Equations (2), (5), and (6), the explicit equation of curvature $u_{2}^{B_{2}\left(s\right)}{|}_{x,y}$ with respect to *β*(*s*) can be obtained

(7) $$u_{2}^{B_{2}\left(s\right)}{|}_{x,y}={\left(K_{1}+K_{2}\right)}_{x,y}^{-1}{\left(R_{Z}^{T}\left(\beta \left(s\right)\right)K_{1}{\bar{u}}_{1}^{B_{1}\left(s\right)}+K_{2}{\bar{u}}_{2}^{B_{2}\left(s\right)}\right)}_{x,y}$$

where the specific value of *β*(*s*) can be obtained by solving the following differential Equation (20)

(8) $$\ddot{\beta }\left(s\right)=\left(1+v\right)\left\|{\bar{u}}_{1}^{B_{1}\left(s\right)}\right\|\left\|{\bar{u}}_{1}^{B_{2}\left(s\right)}\right\|\sin\beta \left(s\right)$$

where *ν* is the Poisson’s ratio of the tube. The boundary condition of (8) is expressed as

(9) $$\beta \left(0\right)=\theta_{1}\left(0\right)-\theta_{2}\left(0\right)$$

(10) $$\dot{\beta }\left(L\right)=\mu^{\prime }\left\|M\right\|\left(1/k_{2z}-1/k_{1z}\right)$$

where *μ′* represents the coefficients related to the friction coefficient, the arm of the concentrated force action, and the radius of the concentric tube robots. Its magnitude can be estimated from experimental data.

### 3.2. Clearance Analysis and Kinematic Modeling

The forward kinematic model of the fitted segment is discussed only in the *B*_2_(*s*) coordinate system. Since the curvature after concentric tube robot fit is a function of the variation along the arc length, the classical assumption of constant curvature no longer applies. The infinitesimal method will be used in this paper. Assuming that the cross-section of each infinitesimal segment has the same curvature, the total arc length of the first section of the concentric tube robot is *L*_1_. The concentric tube robot is divided into *N* sections, and the length of each section is Δ*L*_1_

(11) $$\Delta L_{1}=\frac{L_{1}}{N}$$

The *j*-th segment of the concentric tube robot has the curvature $u_{ij}^{B_{i}\left(s\right)}{|}_{x,y}$ in every cross-section. Its magnitude is taken as the average of the curvatures at the initial and end points of the *j*-th segment. For convenience, the following omits the labeling of the coordinate system. For example, $u_{ij}{|}_{x,y}$ denotes the curvature of the *j*-th infinitesimal segment of the *i*-th concentric tube of the concentric tube robots in the *B*_2_(*s*) coordinate system along the *x* and *y* direction. In addition, since the curvature of the inner and outer tubes in the mating segment is the same, only different representations are available in different coordinate systems, and the following modeling is limited to the inner tube in the *B*_2_(*s*) coordinate system.

**Figure 3:**
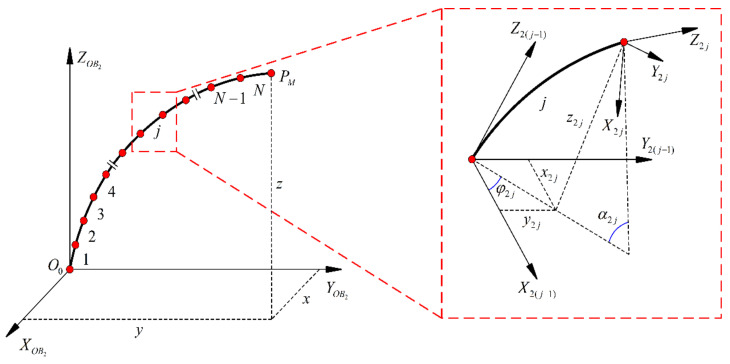
Schematic diagram of the segmentation.

As shown in Figure 3, due to the twisting effect, the *j*-th segment generates a rotation angle *φ*_2*j*_ around the *z*-axis with respect to the (*j* − 1)-th segment.

(12) $$\varphi_{2j}=\tan^{-1}\frac{u_{2j,y}}{u_{2j,x}}$$

The center angle of the *j*-th segment of the concentric tube robots in its bending plane can be expressed as

(13) $$\alpha_{2j}=\left\|u_{2j}\right\|\Delta L$$

The concentric tube robots can be modeled using the DH method. The tip position of the *j*-th segment relative to the *j* − 1 segment endpoint can be obtained from the following equation

(14) $${}_{\;}^{j-1}T_{j}=\left[\begin{array}{cc} R_{z}\left(\varphi_{2j}\right) & 0\\ 0 & 1 \end{array}\right]\left[\begin{array}{cc} R_{y}\left(\alpha_{2j}\right) & X_{j}\\ 0 & 1 \end{array}\right]\left[\begin{array}{cc} R_{z}\left(-\varphi_{2j}\right) & 0\\ 0 & 1 \end{array}\right]$$

(15) $$X_{j}=\left[\begin{array}{c} \left(1-c\alpha_{2j}\right)/\left\|u_{2j}\right\|\\ 0\\ s\alpha_{2j}/\left\|u_{2j}\right\| \end{array}\right]$$

The c*α*_2*j*_ in Equation (15) denotes cos*α*_2_, and s*α*_2*j*_ denotes sin*α*_2*j*_. (*R_z_, R_y_*) ∈ *R*^3^ represents the rotation matrix around the *z*-axis and *y*-axis.

The transformation matrix of the inner tube in the fit segment can be obtained by multiplying the transformation matrix of *N* segments together

(16) $${}^{O^{\prime }}T_{M}=\prod_{j=1}^{N}{}^{j-1}T_{j}$$

Despite the inner tube being circular, there is still a circular curve in the unmatched part of the inner tube. The central angle of the inner tube is $\alpha_{2}=u_{2}L_{2}$, in which *L*_2_ is the total length of the inner tube. The transformation matrix can be obtained as follows

(17) $${}^{O}T_{E}=\left[\begin{array}{cc} R_{z}\left(\theta_{2}\right) & 0\\ 0 & 1 \end{array}\right]{}^{O^{\prime }}T_{M}\left[\begin{array}{cc} R_{z}\left(\Delta \theta \right) & 0\\ 0 & 1 \end{array}\right]\left[\begin{array}{cc} R_{y}\left(\delta \right) & 0\\ 0 & 1 \end{array}\right]\left[\begin{array}{cc} R_{y}\left(\alpha_{2}\right) & X_{2}\\ 0 & 1 \end{array}\right]$$

(18) $$X_{j}=\left[\begin{array}{c} \left(1-c\alpha_{2}\right)/\left\|{\bar{u}}_{2}\right\|\\ 0\\ s\alpha_{2}/\left\|{\bar{u}}_{2}\right\| \end{array}\right]$$

Δ*θ* is a correction term for the additional rotation angle of the inner tube at the *P_M_* point caused by the friction between the inner and outer tubes. Its magnitude can be expressed as Δ*θ = θ*_2_ *+ θ*_1_ − *β*. The positive or negative of each element in Δ*θ* is determined according to the relative rotation direction of the two tubes.

**Figure 4:**
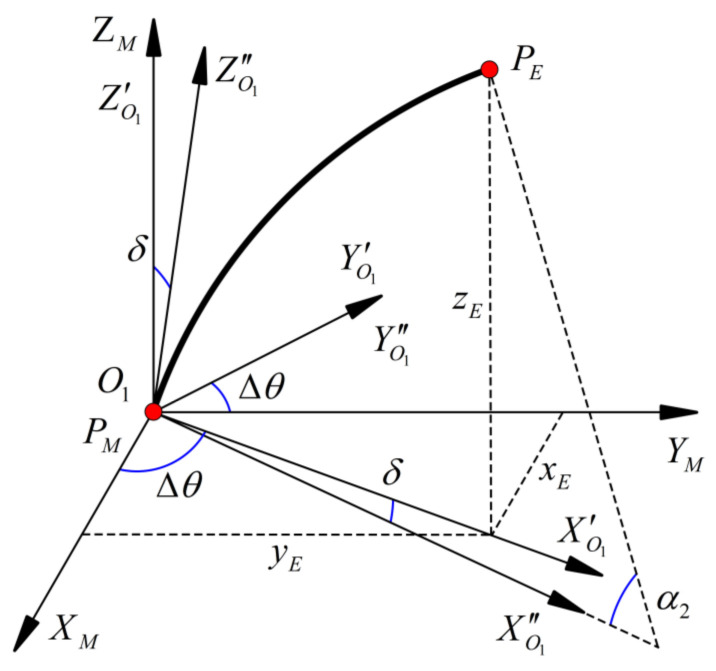
Second segment tubing coordinate system.

As shown in Figure 4, *δ* indicates the deflection of the inner tube at *P_M_*, and is caused by the centerline of the inner and outer tubes not being in the same line. This is due to the clearance between the inner and outer tubes and the effect of deformation. The correction can be completed by assuming that the deflection direction is the bending direction of the inner tube. The value of *δ* can be derived from the experimental data, and we find that the maximum value of the rotation angle occurs at the relative rotation angle of 205°.

According to Equation (17), the coordinates of the endpoint *P_E_* can be obtained.

(19) $$\begin{array}{c} x_{E}={}^{O}T_{E}\left(1,4\right)\\ y_{E}={}^{O}T_{E}\left(2,4\right)\\ z_{E}={}^{O}T_{E}\left(3,4\right) \end{array}$$

The inverse kinematics can be solved by the Levenberg-Marquardt (LM) algorithm [31]. This algorithm can solve the redundancy problem in the inverse kinematic model of concentric tube robots more effectively by adding a damping operator to the iterative formulation.

The LM algorithm can be obtained as follows

(20) $$q_{k+1}=q_{k}+H_{k}^{-1}g_{k}$$

(21) $$H_{k}=J_{k}^{T}W_{E}J_{k}+W_{N}$$

The kinematic model based on the LM algorithm was built in MATLAB.

## 4. Experimental Validation and Analysis of Results

In this section, experiments are conducted to validate the proposed model. In addition, the new model is compared with the original model, which does not consider clearance, friction, and torsion. Concentric tubes made of nylon are created by 3D printing. The tubes are assembled into a straight metal tube. Diamond-shaped cutouts are machined in the walls of the inner and outer tubes to reduce the stiffness. The outer tube’s diamond-shaped cutout is twice the size of the inner tube to balance the stiffness of the tubes. The difference between the outer tube’s inner diameter and the inner tube’s outer diameter is 0.4 mm. Kinematic experiments conducted below demonstrate that a clearance of less than 0.5 mm significantly affects the end path of the concentric tube robots.

### 4.1. Experimental Setup

**Figure 5:**
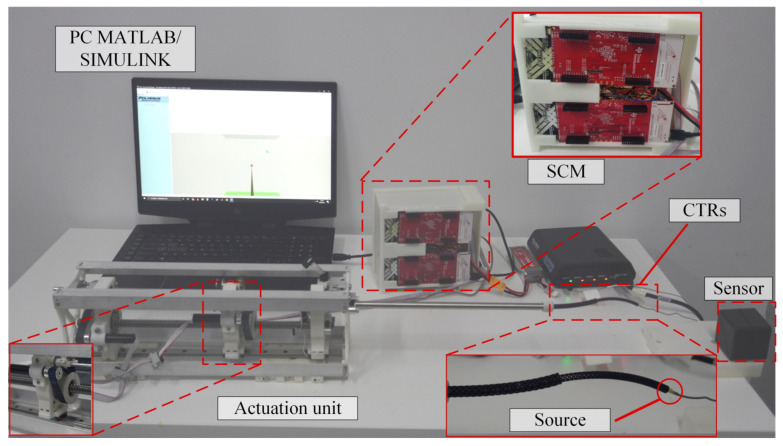
Experimental platform.

The experimental platform designed in this paper is shown in Figure 5. It consists of concentric tube robots, a driver, a controller, an upper computer, and a position measurement unit. The driver consists of four DC servo motors and a transmission mechanism. Of these motors, two control the rotation of two tubes, and the other two control the feeding of the two tubes. The angular positioning error of the servo motor is less than 0.5°, allowing for precise position control. The position measurement unit includes an electromagnetic wave source, a receiver, and a data collector. The receiver obtains the tip position and angle of the concentric tube robots by detecting the electromagnetic wave emission source at the tip of the concentric tube robot. In each experiment, a particular sampling frequency is used to collect the tip position data of the concentric tube robot. The position data are also sent to MATLAB to generate the motion path of the tip.

**Figure 6:**
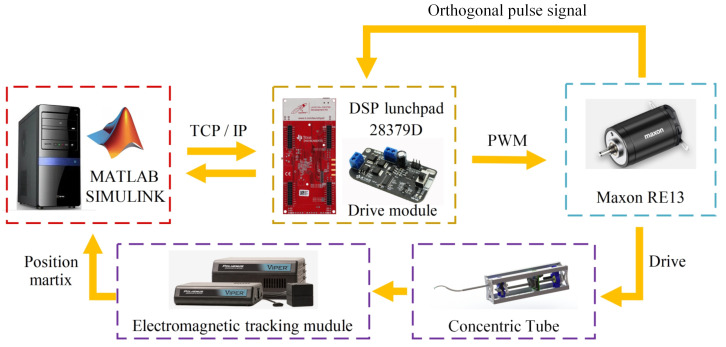
Control system flow.

A closed-loop driver for the servo motor of the robot system was constructed on the host computer using MATLAB/Simulink. The input variables include the rotation angles *θ*_1_ and *θ*_2_, and the translation variables *l*_1_ and *l*_2_. Figure 6 shows the control system logic diagram.

The parameters of this experimental design are shown in Table 1.

### 4.2. Parameter Identification

In order to calculate the forward kinematic path of the improved model, it is necessary to collect data and determine the stiffness of the inner and outer tubes. Different weights are mounted at the end of the tube. The sensors receive the tip position data of the concentric tube robot before and after hanging the weights. The unit load method is used to calculate the inner and outer tube stiffness.

In order to measure the deflection angle of the centerline of the tubes, two sensors are arranged at the connection of the inner and outer tubes. The sensors are fixed to the inner wall of the inner tube and the outer wall of the outer tube to ensure that the two electromagnetic wave sources are facing the same direction as the centerline of the inner and outer tubes. When the concentric tube robot starts to move, the measurement device records the inner and outer tubes’ elevation angle, azimuth angle, and roll angle. These values are input into MATLAB and then calculated as the current deviation angle of the centerline of the two tubes. The initial positions of the sensors are parallel. Clearance data can be obtained when the two tubes are rotated relatively.

### 4.3. Experiment with the Tip Position of the Feed Motion

In order to verify the calculation accuracy of the improved model for the linear feed of the concentric tube robot, prediction experiments of the tip position during linear feeding of the inner tube were conducted. The inner and outer tubes of the concentric tube robot are retracted into a rigid metal tube. The current location is set as the initial location. The inner tube is linearly fed until the end of the inner tube feeding process. During this time, the concentric tube robot’s tip position is transmitted by an electromagnetic wave emission source at a specific frequency. The collected data are transmitted to a PC and recorded in real-time. The tip position of the concentric tube robot is obtained.

**Figure 7:**
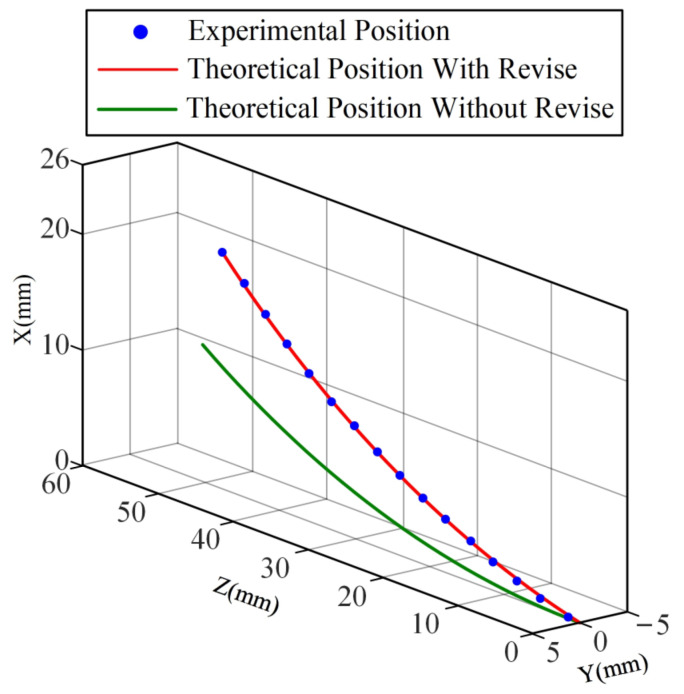
Prediction results of different models for the tip position of the feed path.

The end feed path is entered into the same coordinate system as the path predicted by the improved kinematic model and the path predicted by the previous model. Results are shown in Figure 7. The path predicted by the previous model has a significant deviation from the actual path. The error in the end position predicted by the previous model increases with the feed of the inner tube. Additional deflection of the inner tube induced by the clearance is the cause of this phenomenon. With a feed of 50 mm in the z-axis direction, the targeting position error of the previous model is close to 10 mm. It can be seen that the previous model cannot meet the needs of high precision, such as minimally invasive surgery.

**Figure 8:**
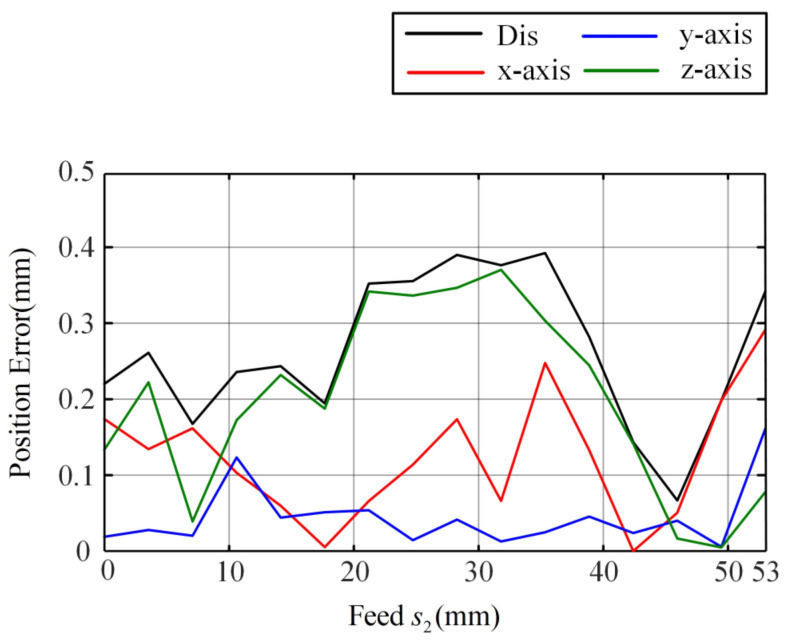
Improved model feed experimental position error.

Due to the consideration of the deflection angle caused by the clearance between tubes in the concentric tube robot feed, the prediction result of the improved model for the path of the tip of the tube is very close to the experimental results. It can accurately predict the tip position during the whole process of inner tube feeding. Figure 8 shows the positioning errors of the improved model, where the maximum error is 0.38 mm in the x direction, 0.15 mm in the y direction, and 0.36 mm in the z direction, and the maximum error in space is 0.39 mm. This result demonstrates the excellent prediction ability of the improved model.

### 4.4. Rotational Motion Tip Position Experiment

**Figure 9:**
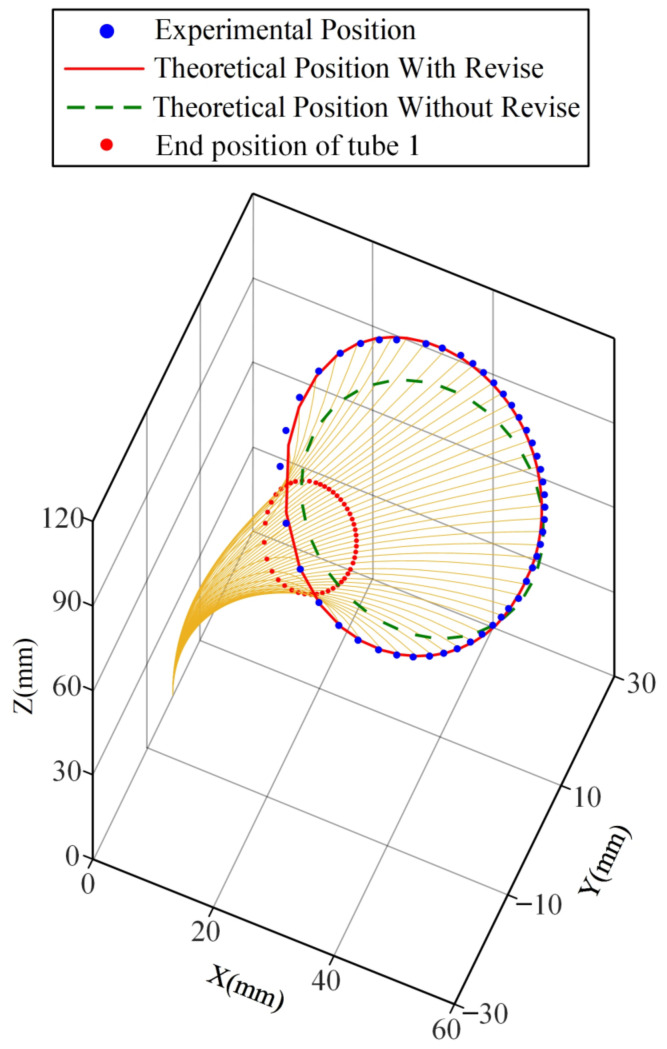
Comparison of the inner tube rotation path of the improved model and the previous model.

**Figure 10:**
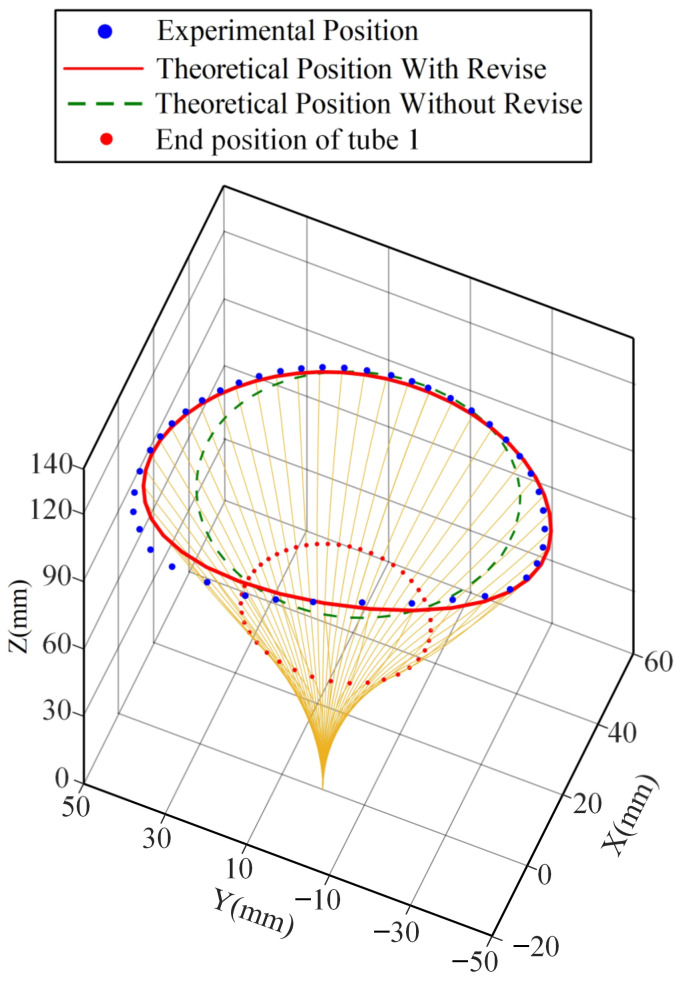
Comparison of the outer tube rotation path of the improved model and the previous model.

In order to verify the predictive ability of the improved model concentric tube robot for tip rotation path, two rotation experiments were conducted. As a first step, the outer tube is fixed, and the inner tube rotated. The position at the beginning of the rotation is set as the initial point. In the same way as the feeding experiment measurement, the tip position data are collected by the same electromagnetic sensor during rotation, and the data during one rotation of the inner tube of the concentric tube robot are recorded at a particular sampling frequency. After the position information is sent to the computer, the data are integrated and inputted into MATLAB and compared with the theoretical position. Figure 9 and Figure 10 show the results of the rotation path for inner and outer tube rotation, respectively.

**Figure 11:**
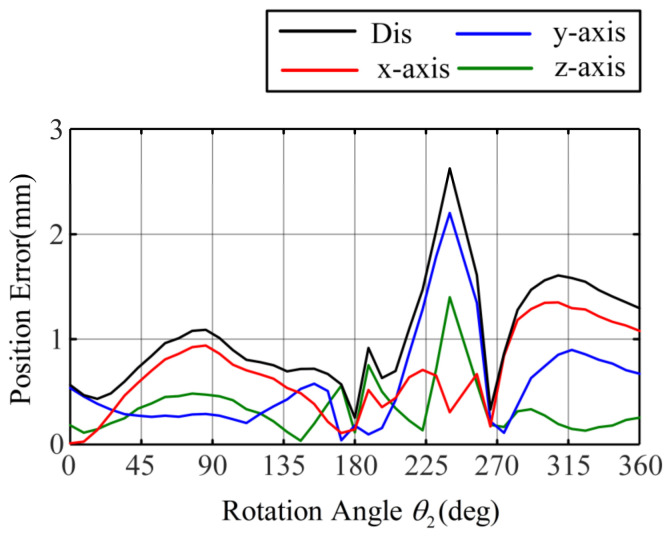
Error of the rotating tip position of the inner tube of the improved model.

**Figure 12:**
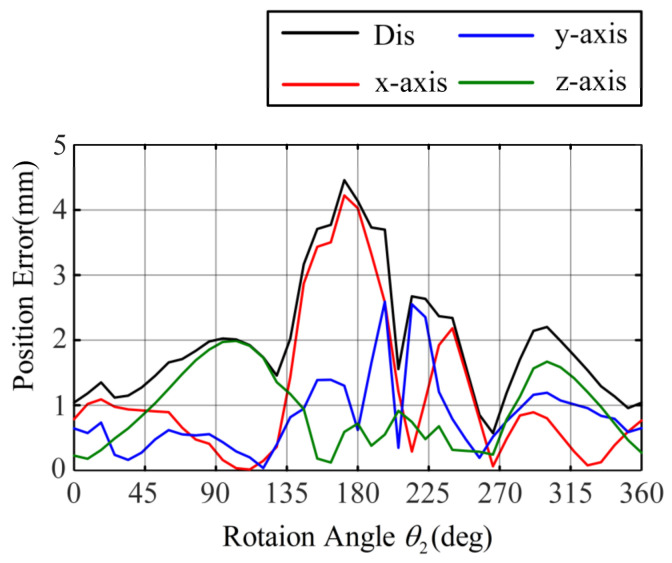
Improved model appearance rotating tip position error.

Considering clearance, friction, and torsion, the improved model still accurately predicts the path of the concentric tube tip position when the friction and torsion effects are significant. Figure 11 and Figure 12 show the tip position error of the inner tube and outer tube of the improved models. The maximum error of the inner tube is 2.6 mm, with an average error of 1.02 mm; the maximum error of the outer tube is 4.5 mm, with an average error of 1.96 mm. The average error of the traditional model in the inner tube rotation and outer tube rotation experiments reach 4.2 mm and 6.7 mm, respectively.

The predictive ability of the improved model is significantly better than that of the traditional model, which can better fit the path of the end of the concentric tube robot when there is a twisting angle between the two tubes. It proves the high accuracy of the improved model.

The experimental results of the feed and rotation experiments are summarized in the following Table 2.

## 5. Conclusions and Future Work

Compared with other continuum robots, concentric tube robots have the advantage of small size. The existing kinematic models of concentric tube robots often neglect clearance, friction, torsion, etc. In order to predict the tip position of concentric tube robots accurately and reduce the tip position error in practical applications, this paper designs a modeling method. This method considers the effects of clearance and friction to accelerate the kinematic modeling. In this paper, by integrating the infinitesimal torsion of the concentric tube robots, the positioning error caused by torsional twisting in the curved portions of the tubes is reduced. We establish a mechanics-based kinematic model and consider the impact of clearance on concentric tube robots as well as the additional deflection of the tube. Then an experimental platform is built, and kinematic experiments are conducted and compared with the kinematic model. The maximum error of the feeding experiment is less than 0.4 mm, and the average error of both inner and outer tube rotation experiments is less than 2 mm. The experimental results show that the improved kinematic model accurately predicts the tip position of the concentric tube robots successfully, with much lower error than the traditional model. Existing modeling methods considering clearance, friction, and torsion have complicated steps. By simplifying the forward kinematics algorithm, the improved method developed in this paper provides a quicker modeling process. It provides a feasible solution for the real-time control of concentric tube robots. In the future, the prototype controlled using the improved model will be subjected to animal experiments to verify the stability and reliability of the improved kinematics model.

## Figures and Tables

**Table 1:** Concentric tube basic parameters.

Design Parameters	Outer Diameter/mm	Inner Diameter/mm	Radius of Curvature/mm	Total Arc Length/mm	Poisson’s Ratio
Tube 1	6.8	4.9	97	74	0.41
Tube 2	4.5	2.7	98.5	114	0.41

**Table 2:** Experimental results.

Experiment	Feed Motion	Rotational Motion (Inner Tube)	Rotational Motion (Outer Tube)
Average error (mm) (improved model)	0.2	1.02	1.96
Maximum error (mm) (improved model)	0.39	2.6	4.5
Average error (mm) (traditional model)	4.95	4.2	6.7
Maximum error (mm) (traditional model)	9.7	5.9	12.1

## Data Availability

Not applicable.
